# Barium Chloride-Induced Cardiac Arrhythmia Mouse Model Exerts an Experimental Arrhythmia for Pharmacological Investigations

**DOI:** 10.3390/life14081047

**Published:** 2024-08-22

**Authors:** Mengting Zeng, Liyue Huang, Xiaohui Zheng, Lebin Weng, Ching-Feng Weng

**Affiliations:** 1 Functional Physiology Section, Department of Basic Medical Science, Xiamen Medical College, Xiamen 361023, China; 18859868050@163.com (M.Z.); hly0915@163.com (L.H.); 201500010365@xmmc.edu.cn (X.Z.); wlb@xmmc.edu.cn (L.W.); 2 Institute of Respiratory Disease, Department of Basic Medical Science, Xiamen Medical College, Xiamen 361023, China; 3 LEADTEK Research, Inc., New Taipei City 235603, Taiwan

**Keywords:** BaCl_2_-induced, cardiac arrhythmia, intraperitoneal, sudden cardiac death, electrocardiogram

## Abstract

Aim: Cardiac arrhythmias are among the most important pathologies that cause sudden death. The exploration of new therapeutic options against arrhythmias with low undesirable effects is of paramount importance. Methods: However, the convenient and typical animal model for screening the potential lead compound becomes a very critical modality, particularly in anti-arrhythmia. In this study, mice were intraperitoneally (i.p.) injected with BaCl_2_, CaCl_2_, and adrenaline to induce arrhythmia, and simultaneously compared with BaCl_2_-induced rats. Results: Electrocardiogram (ECG) showed that the majority of mice repeatedly developed ventricular bigeminy, ventricular tachycardia (VT), and ventricular fibrillation (VF) after BaCl_2_-injection as seen in rats. The ECG of mice developed ventricular bigeminy and VT after CaCl_2_ and AT after adrenaline i.p. injection. Additionally, acute cardiac arrhythmia after BaCl_2_ i.p. injection could be reverted by drugs (lidocaine and amiodarone) administration. Additionally, the different routes of administration for various chemical-induced arrhythmia in both mice and rats were also retrieved from PubMed and summarized. Comparing this approach with previous studies after the literature review reveals that arrhythmia of BaCl_2_-induced i.p. mice is compatible with the induction of other routes. Conclusions: This study brings an alternative experimental model to investigate antiarrhythmic theories and provides a promising approach to discovering new interventions for acute arrhythmias.

## 1. Introduction

Arrhythmia refers to abnormalities in the frequency, rhythm, site of origin, conduction velocity, and order of excitation of cardiac impulses [1]. Arrhythmia, including both atrial and ventricular arrhythmias, represents one of the most common cardiovascular disorders, interfering with daily life and even leading to sudden cardiac death (SCD). Cardiac arrhythmia is a common cardiovascular disease that leads to considerable economic burdens to society. SCD from arrhythmias is a leading cause of mortality accounting for 30% of all deaths [2]. Cardiac arrhythmias and SCD are significant global public health challenges. Ventricular arrhythmias can be provoked by various conditions/diseases with coronary artery disease being most common (particularly during or post-myocardial infarction), but also by any structural heart disease, affecting the ventricles, electrolyte imbalances, metabolic and endocrine disturbances, heart attacks, infection, drugs, etc. Antiarrhythmic drugs (AADs) and antiarrhythmic agents are prescribed for the termination of atrial and ventricular arrhythmias (acute cardioversion) and/or prevention of arrhythmia recurrence (long-term maintenance of normal sinus rhythm). In clinical practice, anti-arrhythmic agents contain at least five groups such as 1. sodium channel blockers, 2. beta-blockers, 3. potassium channel blockers, 4. calcium channel blockers, and 5. other agents that are applied to treat patients [3]. Unexpectedly, anti-arrhythmics might cause several adverse side effects, including constipation, dizziness, headache, nausea, tinnitus, and worsening of asthma, all of which may limit patient compliance/treatment compliance. Many patients are referred for additional interventional treatment (mostly catheter ablation or/and implantable cardioverter-defibrillator implantation). To meet this demand, any new research about mechanisms of origination and treatment including the search for new medicines with fewer adverse effects of ventricular tachycardia is valuable for clinicians.

Animal models of arrhythmia, essential prerequisites for the innovation of novel anti-arrhythmic drugs, focus on various specific inducers to develop different types of experimental animal models for specific types of anti-arrhythmia drug discovery. Some experimental models of cardiac arrhythmia, variously caused by adrenaline in rats, caused by barium in non-narcotized rabbits, caused by strophanthin (Spt) in guinea pigs, or caused by aconitine and calcium in rats, have been successfully developed to evaluate isoteolin manifested antiarrhythmic activity [4]. Numerous experimental models of rodent arrhythmia, developed in guinea pigs, mice, and rats by using the adrenaline- and barium chloride (BaCl_2_)-induced arrhythmia, have been successfully applied to investigate the antiarrhythmic activity of the chemical MG-1, synthesized by aminolysis of 1-(beta, gamma-epoxypropyl)-2-pyrrolidinone and N-phenylpiperazine [5]. One report has illustrated that arrhythmic models of guinea pigs, mice, rats, and rabbits were developed using chloroform (CHCl₃), adrenaline, Spt-K, and BaCl_2_ to explore whether *Cinnamomum migao* could reduce the incidence of ventricular fibrillation (VF) caused by CHCl₃ in mice and the ventricular tachycardia (VT) induced by adrenaline in rabbits, delay the onset time of this arrhythmia, raise the arrhythmic doses of Spt-K in guinea pigs, and moderate the incidence of some arrhythmia caused by BaCl_2_ in rats by slowing down their heart rate [6]. Nineteen target derivatives are evaluated for their antiarrhythmic potential in the mouse model of CHCl₃-induced VF, and five of the derivatives are further investigated in the rat model of BaCl₂-induced arrhythmia [7]. Water-soluble compounds containing diphenylhydantoin basic derivatives have shown strong antiarrhythmic properties in adrenaline-induced arrhythmia, while diphenylhydantoin basic derivatives diphenyl-imidazolidine hydrochloride have exhibited the highest antiarrhythmic activity in the BaCl_2_ arrhythmia model [8]. A series of aminoalkanolic derivatives of xanthone with high affinity for β1-adrenoceptors has been assessed for antiarrhythmic activity in the ischemia–reperfusion isolated hearts, as well as in BaCl_2_- and adrenaline-induced arrhythmia [9].

In a rat model, the inducers (adrenaline, BaCl_2_, and CaCl_2_) are typically injected via the caudal vein and the internal jugular vein to provoke ventricular arrhythmias [7,10]. However, the technique of intravenous injection is not ordinarily easy to accomplish and causes data inconsistency due to the limitation of individual variation. In this study, we aimed to develop a convenient and reliable mouse arrhythmia as an experimental model for new antiarrhythmic drug discovery, in which mice were intraperitoneally (i.p.) injected with BaCl_2_ to induce arrhythmia when simultaneously compared to BaCl_2_-induced rats.

## 2. Materials and Methods

### 2.1. Animal Care

All the experimental procedures were in accordance with the guidelines published by the National Institutes of Health (Guide for the Care and Use of Laboratory Animals, 8th edition) and they fulfilled the ARRIVE guidelines. Animal experiments followed the “Guide for the Care and Use of Laboratory Animals” of Xiamen Medical College and were approved by the Animal Ethics Committee of the Medical College (Approved protocol ID SYXK 2018-0010). This study received financial support from Xiamen Medical College Research Grant (Xiamen Medical College: K2019-01 for Ching-Feng Weng; K2020-07 for Xiaohui Zheng). The evaluations of experimental animal care are periodically examined according to Laboratory Animals—Guidelines for Ethical Review of Animal Welfare (GB/T 35892-2018) [11].

Male 6-week-old ICR mice (22 ± 3 g Bwt) and Sprague Dawley rats (150 ± 30 g Bwt) were obtained from Hangzhou Medical College (Zhejiang, China), and housed at room temperature (22 ± 2 °C) and humidity (50 ± 10%). The 12/12 h light/dark (6 a.m.–6 p.m.) cycle was maintained throughout the entire study. Mice had free access to diet (rodent feed 1022, Beijing HFK Bioscience Co., Ltd., Beijing, China) and tap water ad libitum.

### 2.2. BaCl_2_-Induced Ventricular Arrhythmias in Mice and Rats

Sprague Dawley rats and ICR mice were anesthetized by 5% isoflurane gas in the inhale chamber with vaporizer (rodent gas anesthesia machine R583S, RWD Life Science Co., Ltd., Shenzhen, China) and retained in 2% isoflurane during the whole experimental procedure. The methods for mouse and rat anesthesia were as described in McGill Standard Operating Procedure (SOP) (#110 for mouse and #111 for rat).

**Figure 1:**
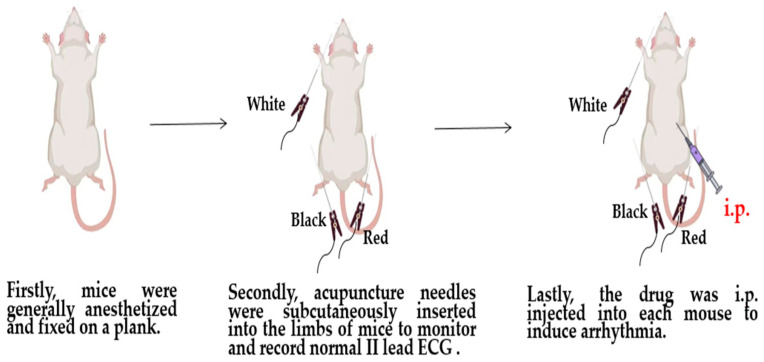
The experimental workflow of barium chloride-induced cardiac arrhythmia mouse and rat models.

Mice were randomly divided into 4 groups including an NS group and 3 tested groups of BaCl_2_, CaCl_2_, and adrenaline (Changzhou Yuanda Pharmaceutical Chemical Co., Ltd., Changzhou, China). Firstly, mice were generally anesthetized, as mentioned above, and fixed on a plank. Secondly, acupuncture needles were subcutaneously inserted into the limbs of mice and rats to monitor and record normal lead II electrocardiogram (ECG) using the BL-420I biological function experiment system (Techman Inc., Chengdu, China) under 2% isoflurane anesthesia. Then, 0.8% BaCl_2_ (0.08 mg/kg Bwt), 1% CaCl_2_ (75 mg/kg Bwt), and 0.002% adrenaline (150 ug/kg Bwt) were intraperitoneally (i.p.) injected into each mouse to induce arrhythmia, respectively. In rats, 0.8% BaCl_2_ (0.16 mg/kg Bwt) was i.p. injected to induce arrhythmia as a control. The surface ECG of mice and rats was continuously recorded, respectively (Figure 1).

### 2.3. Post-Treatment (Rescue) of BaCl_2_ Induction in Mice

Twelve ICR mice were randomly divided into (1) lidocaine group and (2) amiodarone group. Firstly, mice were generally anesthetized and fixed on a plank. Secondly, acupuncture needles were subcutaneously inserted into the limbs of mice to monitor and record normal II lead ECG using the BL-420I biological function experiment system (Techman Inc.) under 2% isoflurane anesthesia. Then, 0.8% BaCl_2_ (0.08 mg/kg Bwt) was i.p. injected into each mouse to induce arrhythmia. When mice showed ventricular bigeminy or ventricular tachycardia (VT), 5% lidocaine (500 mg/kg Bwt, Shanghai Zhaohui Pharmaceatical Co., Ltd., Shanghai, China) and 0.6% amiodarone (15 mg/kg Bwt, Sanofi Hangzhou Pharmaceutical Co., Ltd., Hangzhou, China) were i.p. injected immediately. The surface ECG of mice was continuously recorded.

### 2.4. Statistical Analysis

The in vivo data are expressed as means ± SEMs. The results were carried out by using a one-way analysis of variance (ANOVA) for statistical comparisons among treatments. The means within each column followed by different letters are significantly different at *p* < 0.05 according to the post hoc Tukey’s test.

## 3. Results

### 3.1. BaCl_2_ Induction Arrhythmia versus Adrenaline/CaCl_2_-Induced Arrhythmia

**Figure 2:**
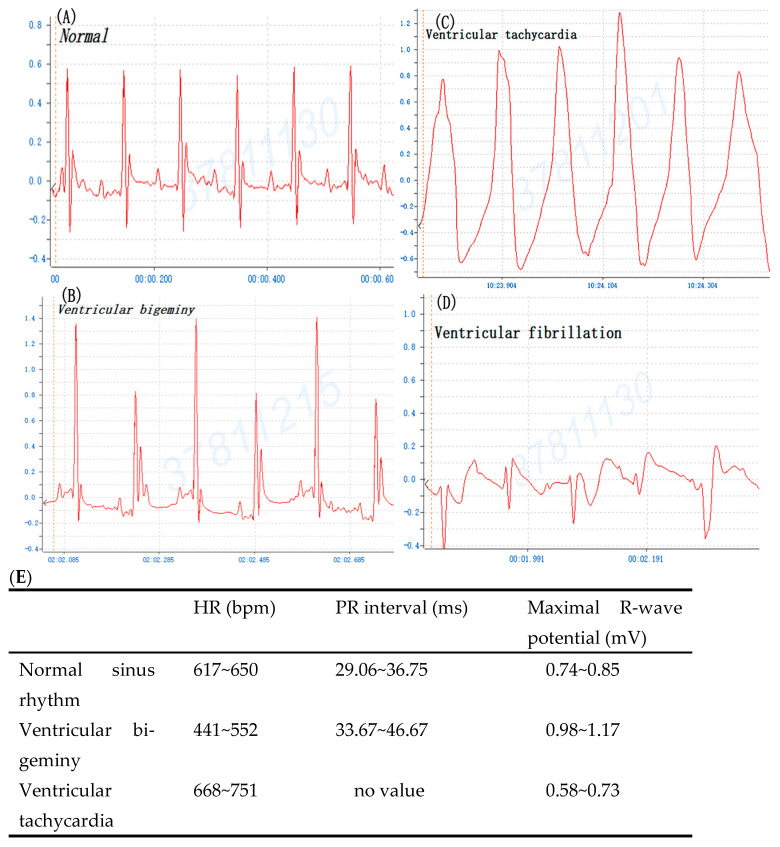
The typical profile of mouse cardiogram prior to and after barium chloride (BaCl_2_, 0.08 mg/kg Bwt) induction. (**A**) Normal sinus rhythm, (**B**) ventricular bigeminy, (**C**) ventricular tachycardia, and (**D**) ventricular fibrillation. (**E**) Alterations of heartbeat (bpm), PR interval (ms), and Maximal R-wave potential (mV) after barium chloride (BaCl_2_) induction.

**Figure 3:**
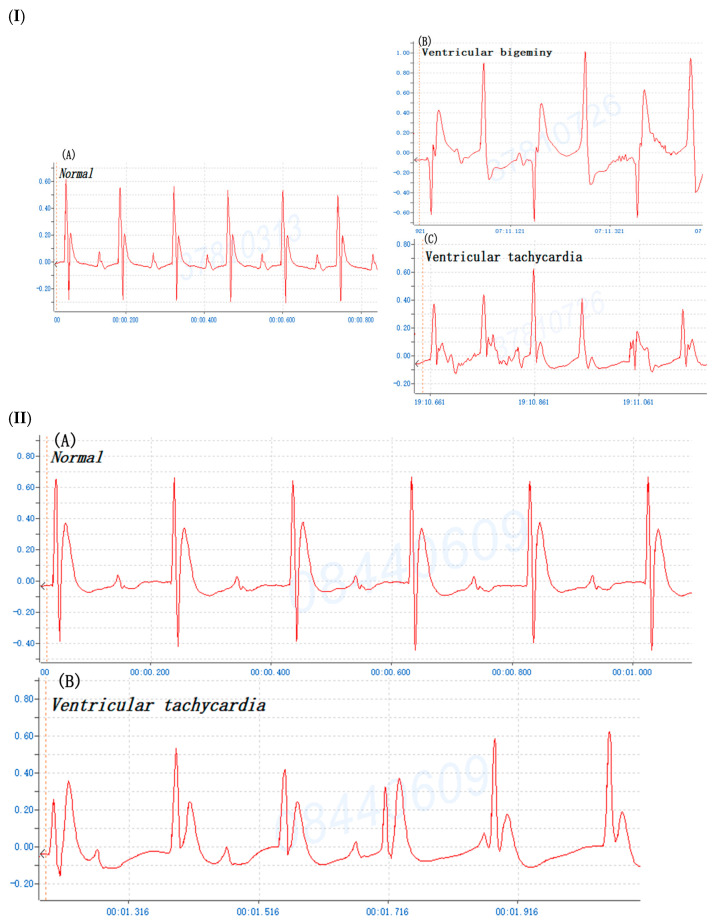
(**I**) The typical profile of mouse cardiogram prior to and after calcium chloride (75 mg/kg Bwt) induction. (**A**) Normal sinus rhythm, (**B**) ventricular bigeminy, and (**C**) ventricular tachycardia. (**II**) The typical profile of mouse cardiogram prior to and after adrenaline (150 ug/kg Bwt) induction. (**A**) Normal sinus rhythm, and (**B**) ventricular tachycardia.

To compare BaCl_2_ induction arrhythmia with adrenaline/CaCl_2_-induced arrhythmia, the mouse was injected i.p. with 0.8% BaCl_2_ (0.08 mg/kg Bwt), 1% CaCl_2_ (75 mg/kg Bwt) and 0.002% adrenaline (150 ug/kg Bwt), respectively. The ECG profile showed that the majority of mice manifested ventricular bigeminy, VT, and VF after BaCl_2_ injection (Figure 2). Figure 2E details the BaCl_2_-induced alterations in ECG parameters of mice including HR (bpm), PR interval (ms), and Maximal R-wave potential (mV). The appearance of ventricular bigeminy and VT in mice after BaCl_2_-induced PR interval (ms) in the EKG profile showed ventricular bigeminy < VT, while Maximal R-wave potential (mV) was ventricular bigeminy > VT. Moreover, the ECG showed that mice developed VT and ventricular bigeminy after CaCl_2_ (Figure 3I) and VT after adrenaline injection (Figure 3II). The time of arrhythmia appearance in mice after i.p. injection was different for various inducers. Ventricular bigeminy 115 ± 20 s; VT 248 ± 18 s; and VF 343 ± 41s were observed after BaCl_2_ induction; ventricular bigeminy 12.3 ± 3.5 min, VT 27.8 ± 4.2 min after CaCl_2_ induction; and VT 42.7 ± 3.1 min after adrenaline induction (Table 1). These data demonstrated that ventricular arrhythmia can also be induced by CaCl_2_ and adrenaline injection i.p. as those similar to BaCl_2_ induction. BaCl_2_-induced arrhythmia in mice by i.p. injection was repeatedly manifested.

### 3.2. Mouse versus Rat of BaCl_2_-Induced Arrhythmia

**Figure 4:**
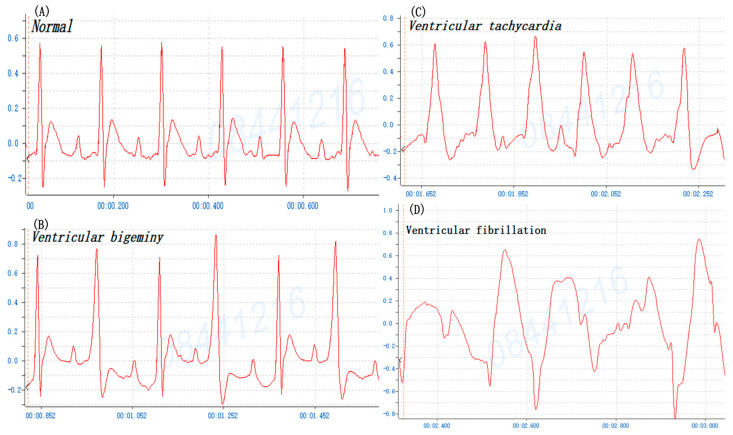
The typical profile of rat cardiogram prior to and after barium chloride (BaCl_2_, 0.16 mg/kg Bwt) induction. (**A**) Normal sinus rhythm, (**B**) ventricular bigeminy, (**C**) ventricular tachycardia, and (**D**) ventricular fibrillation.

Multiple ventricular arrhythmias may be induced following the administration of BaCl_2_ in rats, particularly ventricular premature contraction (VPC) and VT [12,13,14]. To confirm the BaCl_2_ induction arrhythmia in mice is comparable to that seen in rats, the rat was injected i.p. with 0.8% BaCl_2_ solution (0.16 mg/kg Bwt). The profiles of ECG showed that rats also manifested ventricular bigeminy, VT, and VF after BaCl_2_ injection (Figure 4). The data indicate that the ECG profile after BaCl_2_ induction arrhythmia in both mice and rats is analogous except that the heartbeat speed is different. The different administration routes of various chemical-induced arrhythmia in both mice and rats were also reviewed and summarized (Table 2). Comparing the present study with previous studies reveals that arrhythmia of BaCl_2_-induced i.p. mice is comparable to various chemical-induced arrhythmias through different routes of administration, suggesting the BaCl_2_ induction mouse model is appropriate for an experimental model in the investigation of cardiac arrhythmia and analysis of the underlying mechanisms. This mouse model is a sensitive, reproducible, inexpensive, rapid technique for in vivo preliminary screening of antiarrhythmic compounds particularly in the discovery and development of new drugs.

### 3.3. Post-Treatment (Rescue) of BaCl_2_ Induction Arrhythmia in Mice

**Figure 5:**
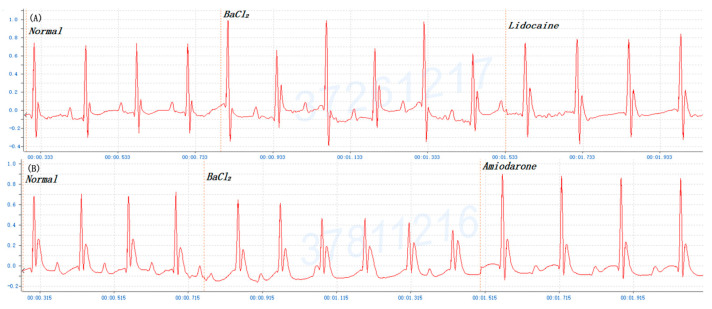
The treatments of mouse cardiogram after barium chloride (BaCl_2_, 0.08 mg/kg Bwt)-induced arrhythmia: (**A**) 0.6% amiodarone (15 mg/kg Bwt), (**B**) 5% lidocaine (500 mg/kg Bwt). This ECG pattern is separately cut from the original profile into three sections: (1) normal sinus rhythm, (2) BaCl_2_, and (3) amiodarone or lidocaine, respectively, for the presentation.

**Figure 6:**
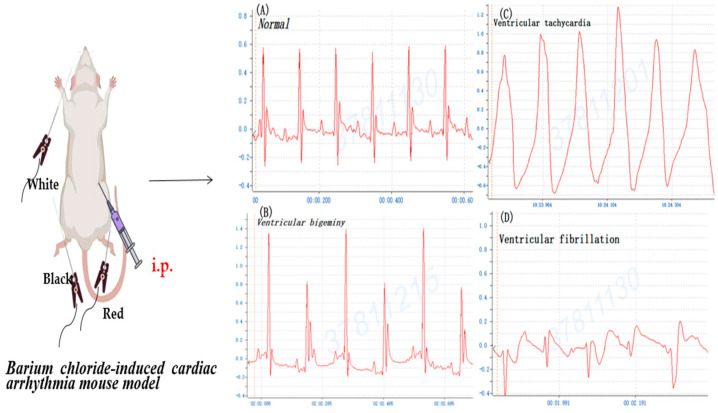
Summary of the profile of cardiac arrhythmia in barium chloride-induced mouse.

Amiodarone, a widely used class III antiarrhythmic drug, also exerts its antiarrhythmic effect through suppression of associated K^+^ channels. Amiodarone is an effective treatment for atrial and ventricular arrhythmias; however, its use is limited by a toxic adverse effect profile. The amiodarone-induced K^+^ channel blockade may result in the prolongation of ventricular repolarization, which finally leads to LQTS, TdP, and VF [38,39]. In clinical practice, amiodarone is used for anti-arrhythmias, especially atrial arrhythmia with duration therapy. Lidocaine is a local anesthetic of the amino amide type and is also used to treat VT. Meanwhile, lidocaine (sodium channel blockers) is administered for the treatment of ventricular arrhythmia with duration therapy. Furthermore, to validate the success of this model as an experimental cardiac arrhythmia, amiodarone and lidocaine were used to rescue mice with BaCl_2_ induction arrhythmia. Once the manifestations of ventricular arrhythmia occurred in mice, 5% lidocaine (500 mg/kg Bwt) and 0.6% amiodarone (15 mg/kg Bwt) were injected i.p. immediately. The data showed that mice with ventricular arrhythmia could be returned to normal sinus rhythm by lidocaine and amiodarone treatments (Figure 5). Four-sixths of mice in the amiodarone group returned to normal ECG, and five-sixths of mice in the lidocaine group returned to normal ECG. This experiment reconfirms that BaCl_2_ induction arrhythmia in mice can be applied as an animal model to discover new compounds for the treatment of cardiac arrhythmia as summarized in Figure 6.

## 4. Discussion

A major challenge remains in arrhythmia research because it can be investigated only in vivo. Various animal species have been used, and several disease models have been developed to study arrhythmias. Numerous animals including cats, dogs, rabbits, pigs, rats, mice, guinea pigs, and zebrafish can be induced by different chemicals such as CHCl₃, Stp, adrenaline, BaCl_2_, and CaCl_2_ through various routes (*iv*, i.p., immersion, inhalation), and these have been developed as arrhythmia experimental models for the investigation of antiarrhythmic theories and the discovery new drugs for acute arrhythmias treatment. The majority of experimental murine models of arrhythmia are induced by intravenous (jugular vein or sublingual vein) drugs or caudal vein injection, which make the operation complicated, necessitating a longer operation time (summarized in Table 2). We note that BaCl_2_ is a simple, fast, and effective drug widely used in the preparation of models for ventricular arrhythmia [7,8,9]. The induction of ventricular arrhythmia by injecting BaCl_2_ into the internal jugular vein has been reported [15]. When comparing glialin with allapinin, the antiarrhythmic activity of glialin is qualitatively analogous to that of allapinine based on the low toxicity of glialin over allapinin, which is due to the presence of glycyrrhizic acid. This result is reported by using rats and guinea pigs through intravenous administration of CaCl_2_, aconitine, BaCl_2_, and Stp [13]. Accordingly, BaCl_2_-induced arrhythmia in mice by i.p. injection is repeatable and is suitable for the exploration of intervention. Both cleistanthin A and cleistanthin B isolated from the leaves of *Cleistanthus collinus* Roxb. (Euphorbiaceae) have been found to have a hypotensive effect and to have no antiarrhythmic effect against BaCl_2_-induced Wistar rats [10]. As shown in Table 2, the BaCl_2_-induced mouse arrhythmia model by i.p. is a fast, easy, and applicable method with intervention (lidocaine, sodium channel blockers, and amiodarone, drugs prolonging the action potential duration).

There are numerous diverse types of arrhythmias comprising atrial fibrillation (AF), atrial flutter, VF, and VT. Ventricular arrhythmias, including VT and VF, are highly associated with SCD [40]. Adrenaline, BaCl_2_, and CaCl_2_ are injected into the caudal vein in rats to induce ventricular arrhythmia [41]. Likewise, we have successfully achieved the antiarrhythmic activity in adrenaline-, CaCl_2_- and BaCl_2_-induced arrhythmia models in mice by i.p. injection. Five-sixths of mice developed ventricular bigeminy, VT, and VF after BaCl_2_ injection (Figure 2). In addition, four-sixths of mice developed ventricular bigeminy and VT without VF after CaCl_2_ injection. In the adrenaline group, four-sixths of mice only developed VT. The ECG data showed successful induction with all mice developing ventricular arrhythmias. Of note, BaCl_2_ is a highly toxic salt and has arrhythmogenic effects by impairing ion channels in cardiomyocytes. Subsequently, BaCl_2_ leads to an increase in Na^+^ and Ca^2+^ influx, which promotes delays after depolarization. Ba^2+^ is a potassium ion (K^+^) channel blocker competing at the K^+^-binding site, which reduces the outflow of K^+^ to produce ventricular arrhythmia [42,43,44]. The downregulation of the inward rectifier potassium (IK1) channel is a hallmark of cardiac hypertrophy and failure. IK1 antagonist chloroquine or BaCl_2_ can largely reverse the cardioprotective effect of zacopride (a selective IK1/Kir2.1 channel agonist) [45,46]. BaCl_2_ causes a significant decrease in acetylcholinesterase, catalase, and superoxide dismutase activities as well as glutathione levels in the heart and lungs of the treated rats. Moreover, the lipid peroxidation in cardiac and lung tissue shows a dose-dependent increase, while advanced oxidative protein product and nitric oxide levels are associated with a significant increase in metallothionein of the BaCl_2_-treated rats. The BaCl_2_-induced heart and lung damage could be due to the interruption of antioxidant defense systems, and the triggering of inflammatory mediators and alterations in the hematological parameters of rats [47].

During the ensuing years, several studies revealed that cultured cells often lack the correct ion channel subunit stoichiometry and subcellular organization found in native cardiac myocytes. While acknowledging that rodents are easily accessible model animals, their electrophysiological characters are profoundly different from those of humans, making the extrapolation of rat studies to humans moderately difficult. Mouse models are superior to cell culture models since intact mouse hearts contain all relevant types of specialized cells including nodal and conduction system cells, endothelial cells, and fibroblasts. In vivo models, and studies evaluating treatments and interventions for arrhythmogenic disease need to take into account the relative and sometimes complex advantages of each species and disease model. Mice also offer human-like cardiac anatomy not seen in simpler animal models such as zebrafish (*Danio rerio*), which lack the right atrium and ventricle. Compared to mouse models, large animal models have heart rates, action potential shapes and durations, ion channel profiles, and intracellular Ca^2+^-handling systems with dynamics that are more similar to those seen in humans [48]. Therefore, large animal models play a key role in preclinical studies, although it remains very difficult and expensive to perform gene-targeting in mouse models by using AAV for example. One previous report in cardiac electrophysiological experiments has found that dog ventricular preparations have the strongest translational value while rat ventricular preparations have the weakest ones. This indicates that the different manifestations in the inhibition of various potassium currents and their effects on repolarization in cardiac ventricular muscle are species-dependent [49]. Chemically induced arrhythmia contains aconitine antagonism, digoxin-induced arrhythmia, strophanthin/ouabain-induced arrhythmia, adrenaline-induced arrhythmia, and calcium-induced arrhythmia, particularly in mice and rats as presented in Table 2. In rats and guinea pigs are treated by *iv* administration of CaCl_2_, aconitine, BaCl_2_, and Stp. According to the observed values of chaos, the highest one is observed in dogs, while rabbits and rats are intermediate and the lowest degree of chaos is observed in humans. Observation of the structure–function organization of the myocardium has shown that fibrillation in humans and animals has a different degree of regularity and diverse values of the chaotic component [50]. Consequently, the measure of QT dispersion from the 2-lead ECG in the rat described in this protocol is different than that calculated from human ECG records. This represents a limitation in the translation of the data obtained from rodents to human clinical medicine. Alternatively, comparing the present study with the previously published information reveals that arrhythmia of BaCl_2_-induced via i.p. mice is compatible with various chemical-induced arrhythmias through different routes of administration.

We consider the potential limitations of using murine models to represent human cardiac physiology more explicitly. The broad availability of transgenic mouse models and the option to generate mice with cell-specific and/or time-dependent regulation of gene expression provides a significant advantage for mice over other small animal species. A major limitation is that their fast heart rate, small heart size, and differential ionic currents do not fully recapitulate human cardiac electrophysiology. According to the ECGs, murine and human models show salient differences, including the occurrence of a pronounced J-wave and a less distinctive T-wave in the murine ECG. Mouse models can resemble human cardiac arrhythmias, although mice differ from humans in cardiac electrophysiology [51]. Therefore, it is important to recognize species differences in cardiac AP waveforms and underlying membrane currents for ventricular cardiomyocytes of humans and mice, which are the result of species-specific expression of ion channels and transporters. Unlike humans, mice and rats have a low AP plateau at ≈−40 mV membrane potential [52]. Furthermore, there are limiting concerns of the BaCl_2_-induced via i.p. mouse arrhythmia model. First, Ba^2+^ is a foreign substance that is not essential for our body and is rarely encountered in life. Second, Ba^2+^, similar to Ca^2+^, can also cause a constriction of smooth muscle, which leads to gastrointestinal symptoms via i.p. injection. Moreover, with the apparent difference in size (anatomical structure) and faster beating rate (four to eight times faster), there are still some other limitations when extrapolating the repolarization findings (action potential shapes and durations) and intracellular Ca^2+^-handling systems with dynamics obtained with the mouse model to the human heart [48,53]. Further, mice present differences in their ion channel profiles when compared to the human heart (i.e., TTX-sensitive sodium channel transcript levels decreased with increasing heart size) [54]. This different expression profile results in different action potential characteristics and pharmacology. While mice with gene knockout are usually used for the model epigenetic or posttranslational modifications associated with acquired arrhythmia conditions, numerous studies have demonstrated using mice for genetic investigations, mechanistic evaluations, or early studies to identify potential drug targets; rabbits for studies on ion channel function, repolarization or re-entrant arrhythmias; and pigs for preclinical translational studies to validate previous findings [55]. Moreover, mouse models offer the opportunity to test and validate disease-causing mechanisms originating outside the cardiovascular system. These concepts may become very critical for the translation of experimental results to the clinical setting. Additionally, mouse models can also be helpful for drug development efforts and to better understand the underlying processes of arrhythmogenesis, and offer the opportunity to perform affordable preclinical evaluation of therapeutic efficacy and safety in vivo and ex vivo in isolated hearts or cardiac myocytes isolated from mutant mice.

## 5. Conclusions

The manifestation of ECG showed that the majority of mice repeatedly developed ventricular bigeminy, VT, and VF after BaCl_2_ i.p. injection. BaCl_2_-induced arrhythmia in mice is comparable with BaCl_2_-induced arrhythmia episodes in rats. Moreover, the ECG showed that mice also developed ventricular bigeminy and VT after CaCl_2_ and VT after adrenaline injection. Acute cardiac arrhythmia after BaCl_2_ i.p. injection could be reverted by lidocaine (IB type, sodium channel blockers) and amiodarone (III type, potassium channel blockers) drug administration. Prospectively, this study brings an alternative experimental model to investigate anti-arrhythmic theories and provides a promising approach to explore new indications of medicine or phytochemicals for the intervention of acute arrhythmias.

## Figures and Tables

**Table 1:** Appeared time of chemical-induced arrhythmia in mice by intraperitoneal injection.

Inducer	Ventricular Bigeminy	Ventricular Tachycardia	Ventricular Fibrillation
0.8% BaCl_2_	115 ± 20 s	248 ± 18 s ***	343 ± 41 s
1% CaCl_2_	12.3 ± 3.5 min	27.8 ± 4.2 min ##	-
0.002% Adrenaline	-	42.7 ± 3.1 min	-

0.8% BaCl_2_ (0.08 mg/kg Bwt); 1% CaCl_2_ (75 mg/kg Bwt); 0.002% adrenaline (150 ug/kg Bwt); -: not found; s: second; Each group, n = 6; ***, *p* < 0.001 (BaCl_2_ vs. CaCl_2_ and adrenaline), one way ANOVA, ##, *p* < 0.01 (CaCl_2_ vs. adrenaline).

**Table 2:** Various routes of chemical-induced arrhythmia in mice and rats.

Animal	Inducer	Route	Manifestation	Reference
Rats	BaCl_2_ BaCl_2_	*iv* *iv*	VF -	[6] [7]
	BaCl_2_ Adrenaline	* iv or po *	-	[9]
	BaCl_2_	Internal jugular vein	-	[15]
	Iso	i.p.	AF	[16]
	BaCl_2_,	Caudal vein	AF,	[17]
	Aconitine	Caudal vein	VF, VT	[18]
	Aconitine	*iv*	-	[19]
	Aconitine	i.p.	-	[20]
	Ach-CaCl_2_	Caudal vein	AF	[21]
	Ach-CaCl_2_	Caudal vein	AF	[22]
	Ach-CaCl_2_	Caudal vein	AF	[23]
	BaCl_2_, CaCl_2_	*iv*	AF	[24]
	Epinephrine	*iv*	VF, VT	[25]
	Epinephrine	Infusion	-	[26]
	BaCl_2_	External jugular veins	VF	[27]
	Epinephrine	*iv*	VT	[28]
	Aconitine	*iv*	-	[29]
	Aconitine	Femoral vein	-	[30]
	Adrenaline, BaCl_2_, CaCl_2_, aconitine	*iv*	-	[31]
	Epinephrine	Caudal vein	Bradyarrhythmia	[32]
	Aconitine	Infusion	VT, VF	[33]
	BaCl_2_	Caudal vein		[17]
	CaCl_2_, BaCl_2_, electric		AF	[24]
	Aconitine, BaCl_2_		VP, VT, VF, CA	[34]
Mice	BaCl_2_	Caudal vein	-	[7]
	Chloroform	Inhalation	VF	[7]
	Chloroform	Inhalation	-	[33]
	Chloroform	Inhalation	VES, VF, VT	[35]
	Aconitine	Infusion caudal vein	VT	[36]
	Carbamyl choline	Jugular vein	AF, AT	[37]
	BaCl_2_	i.p.	VB, VF, VT	Present study

Acetylcholine (Ach), barium chloride (BaCl_2_), calcium chloride (CaCl_2_), isoproterenol (Iso), atrial fibrillation (AF), atrial tachycardia (AT), ventricular tachycardia (VT), ventricular bigeminy (VB), ventricular fibrillation (VF), and ventricular extrasystoles (VES). -: not shown.

## Data Availability

Data are contained within the article.

## References

[1] Kovacs B., Mayinger M., Andratschke N., Saguner A.M. (2022). Stereotactic arrhythmia radioablation: Competitor or adjunct to catheter ablation?. Eur. Heart J..

[2] Green D., Roberts P.R., New D.I., Kalra P.A. (2011). Sudden cardiac death in hemodialysis patients: An in-depth review. Am. J. Kidney Dis..

[3] Saljic A., Heijman J., Dobrev D. (2023). Recent Advances in Antiarrhythmic Drug Therapy. Drugs.

[4] Markov M., Zheliazkov D., Iakimov G. (1990). Vŭrkhu antiaritmichnata aktivnost na izoteolina (IZT) [The anti-arrhythmia activity of isoteolin (IST)]. Eksp. Med. Morfol..

[5] Malawska B., Gorczyca M., Filipek B. (1992). Synthesis, physicochemical and preliminary pharmacological properties of N-[beta-hydroxy-gamma-(N-phenylpiperazinepropyl)]-2-pyrrolidinone. Pol. J. Pharmacol. Pharm..

[6] Sui Y., Qiu D., Xie C., Chen K. (1998). Observation of antiarrhythmic effects of *Cinnamomum migao* H. W. Li on experimental arrhythmia. Zhongguo Zhong Yao Za Zhi.

[7] Wang H., Cheng X., Kong S., Yang Z., Wang H., Huang Q., Li J., Chen C., Ma Y. (2016). Synthesis and Structure-Activity Relationships of a Series of Aporphine Derivatives with Antiarrhythmic Activities and Acute Toxicity. Molecules.

[8] Dylag T., Zygmunt M., Maciag D., Handzlik J., Bednarski M., Filipek B., Kieć-Kononowicz K. (2004). Synthesis and evaluation of in vivo activity of diphenylhydantoin basic derivatives. Eur. J. Med. Chem..

[9] Rapacz A., Sapa J., Bednarski M., Filipek B., Szkaradek N., Marona H. (2014). Antiarrhythmic activity of some xanthone derivatives with β1-adrenoceptor affinities in rats. Eur. J. Pharmacol..

[10] Pytka K., Lustyk K., Żmudzka E., Kotańska M., Siwek A., Zygmunt M., Dziedziczak A., Śniecikowska J., Olczyk A., Gałuszka A. (2016). Chemically Homogenous Compounds with Antagonistic Properties at All α1-Adrenoceptor Subtypes but Not β1-Adrenoceptor Attenuate Adrenaline-Induced Arrhythmia in Rats. Front. Pharmacol..

[11] (2018). Laboratory Animal—Guideline for Ethical Review of Animal Welfare in China.

[12] Liu B., Li S., Su Y., Xiong M., Xu Y. (2014). Comparative study of the protective effects of terfenadine and amiodarone on barium chloride/aconitine-induced ventricular arrhythmias in rats: A potential role of terfenadine. Mol. Med. Rep..

[13] Khisatmutdinova RIu Baschenko NZh Zarudiĭ F.S., Gabdrakhmanova S.F., Makara N.S., Sapozhnikova T.A. (2006). Some aspects of the antiarrhythmic effect of glialin. Eksp. Klin. Farmakol..

[14] Huang W., Wang Y., Cao Y.G., Qi H.P., Li L., Bai B., Liu Y., Sun H.L. (2013). Antiarrhythmic effects and ionic mechanisms of allicin on myocardial injury of diabetic rats induced by streptozotocin. Naunyn Schmiedebergs Arch. Pharmacol..

[15] Al-Khatib S.M., Stevenson W.G., Ackerman M.J., Bryant W.J., Callans D.J., Curtis A.B., Deal B.J., Dickfeld T., Field M.E., Fonarow G.C. (2018). 2017 AHA/ACC/HRS Guideline for Management of Patients with Ventricular Arrhythmias and the Prevention of Sudden Cardiac Death: A Report of the American College of Cardiology/American Heart Association Task Force on Clinical Practice Guidelines and the Heart Rhythm Society. Heart Rhythm..

[16] Chen X., Wan W., Ran Q., Ye T., Sun Y., Liu Z., Liu X., Shi S., Qu C., Zhang C. (2022). Pinocembrin mediates antiarrhythmic effects in rats with isoproterenol-induced cardiac remodeling. Eur. J. Pharmacol..

[17] Zhao Y., Wang X., Chen C., Shi K., Li J., Du R. (2021). Protective Effects of 3,4-Seco-Lupane Triterpenes from Food Raw Materials of the Leaves of *Eleutherococcus Senticosus* and *Eleutherococcus Sessiliflorus* on Arrhythmia Induced by Barium Chloride. Chem. Biodivers..

[18] Joukar S., Ghorbani-Shahrbabaki S., Hajali V., Sheibani V., Naghsh N. (2013). Susceptibility to life-threatening ventricular arrhythmias in an animal model of paradoxical sleep deprivation. Sleep Med..

[19] Runtao G., Guo D., Jiangbo Y., Xu W., Shusen Y. (2011). Oxymatrine, the main alkaloid component of Sophora roots, protects heart against arrhythmias in rats. Planta Med..

[20] Chen X., Guo H., Li Q., Zhang Y., Liu H., Zhang X., Xie K., Zhu Z., Miao Q., Su S. (2018). Protective effect of berberine on aconite-induced myocardial injury and the associated mechanisms. Mol. Med. Rep..

[21] Zou D., Geng N., Chen Y., Ren L., Liu X., Wan J., Guo S., Wang S. (2016). Ranolazine improves oxidative stress and mitochondrial function in the atrium of acetylcholine-CaCl_2_ induced atrial fibrillation rats. Life Sci..

[22] Zhao J., Zhang Q., Zou G., Gao G., Yue Q. (2020). Corrigendum to “Arenobufagin, isolated from toad venom, inhibited epithelial-to-mesenchymal transition and suppressed migration and invasion of lung cancer cells via targeting IKKβ/NFκB signal cascade”. J. Ethnopharmacol..

[23] Feng R., Wan J., He Y., Gong H., Xu Z., Feng J. (2023). Angiotensin-receptor blocker losartan alleviates atrial fibrillation in rats by downregulating frizzled 8 and inhibiting the activation of WNT-5A pathway. Clin. Exp. Pharmacol. Physiol..

[24] Sergeevichev D., Fomenko V., Strelnikov A., Dokuchaeva A., Vasilieva M., Chepeleva E., Rusakova Y., Artemenko S., Romanov A., Salakhutdinov N. (2020). Botulinum Toxin-Chitosan Nanoparticles Prevent Arrhythmia in Experimental Rat Models. Mar. Drugs.

[25] Xue Y.X., Aye N.N., Hashimoto K. (1996). Antiarrhythmic effects of HOE642, a novel Na^+^-H^+^ exchange inhibitor, on ventricular arrhythmias in animal hearts. Eur. J. Pharmacol..

[26] Crawford M.W., Ho D.S., Shams M., Gow R. (2004). Magnesium deficiency alters the threshold for epinephrine-induced arrhythmias during halothane or sevoflurane anesthesia in the rat. J. Cardiothorac. Vasc. Anesth..

[27] Ghasi S. (2008). Piperazine protects the rat heart against sudden cardiac death from barium chloride-induced ventricular fibrillation. Am. J. Ther..

[28] Rabkin S.W. (1992). Dynorphin A (1–13) in the brain suppresses epinephrine-induced ventricular premature complexes and ventricular tachyarrhythmias. Regul. Pept..

[29] Wascher T.C., Dittrich P., Kukovetz W.R. (1991). Antiarrhythmic effects of two new propafenone related drugs. A study on four animal models of arrhythmia. Arzneimittel-Forschung.

[30] Müller B., Wilsmann K. (1982). Effects of the optical isomers of D 600 on cardiovascular parameters and on arrhythmias caused by aconitine and coronary artery ligation in anesthetized rats. J. Cardiovasc. Pharmacol..

[31] Kozlovski V.I., Vdovichenko V.P., Chlopicki S., Malchik S.S., Praliyev K.D., Zcilkibayev O.T. (2004). Antiarrhythmic profile and endothelial action of novel decahydroquinoline derivatives. Pol. J. Pharmacol..

[32] Hoffmann P., Müller S., Zbinden G. (1992). Decrease of epinephrine-induced arrhythmia threshold in ethanol exposed rats. Arch. Toxicol..

[33] Dai S. (1986). Effects of ranitidine and cimetidine on experimentally induced ventricular arrhythmias in anaesthetized rats. Agents Actions.

[34] Yang W., Wang W., Cai S., Li P., Zhang D., Ning J., Ke J., Hou A., Chen L., Ma Y. (2023). Synthesis and In Vivo Antiarrhythmic Activity Evaluation of Novel Scutellarein Analogues as Voltage-Gated Nav1.5 and Cav1.2 Channels Blockers. Molecules.

[35] Brooks R.R., Carpenter J.F., Miller K.E., Maynard A.E. (1996). Efficacy of the class III antiarrhythmic agent azimilide in rodent models of ventricular arrhythmia. Proc. Soc. Exp. Biol. Med..

[36] Dai S., Chan M.Y., Lee S.S., Ogle C.W. (1986). The antiarrhythmic effects of *Sophora flavescens* Ait. in rats and mice. Am. J. Chin. Med..

[37] Wakimoto H., Maguire C.T., Kovoor P., Hammer P.E., Gehrmann J., Triedman J.K., Berul C.I. (2001). Induction of atrial tachycardia and fibrillation in the mouse heart. Cardiovasc. Res..

[38] Wilhelms M., Rombach C., Scholz E.P., Dössel O., Seemann G. (2012). Impact of amiodarone and cisapride on simulated human ventricular electrophysiology and electrocardiograms. Europace.

[39] Taniguchi T., Uesugi M., Arai T., Yoshinaga T., Miyamoto N., Sawada K. (2012). Chronic probucol treatment decreases the slow component of the delayed-rectifier potassium current in CHO cells transfected with KCNQ1 and KCNE1: A novel mechanism of QT prolongation. J. Cardiovasc. Pharmacol..

[40] Coast G.M. (2012). Intracellular Na^+^, K^+^ and Cl^−^ activities in Acheta domesticus Malpighian tubules and the response to a diuretic kinin neuropeptide. J. Exp. Biol..

[41] Kehl S.J., Fedida D., Wang Z. (2013). External Ba^2+^ block of Kv4.2 channels is enhanced in the closed-inactivated state. Am. J. Physiol. Cell Physiol..

[42] Rowley C.N., Roux B. (2013). A computational study of barium blockades in the KcsA potassium channel based on multi-ion potential of mean force calculations and free energy perturbation. J. Gen. Physiol..

[43] Ghasi S., Mbah A.U., Nze P.U., Nwobodo E., Ogbonna A.O., Onuaguluchi G. (2009). Interventional Role of Piperazine Citrate in Barium Chloride Induced Ventricular Arrhythmias in Anaesthetized Rats. Biomed. Res..

[44] Parasuraman S., Raveendran R., Selvaraj R.J. (2011). Effects of cleistanthins A and B on blood pressure and electrocardiogram in Wistar rats. Z. Naturforschung C.

[45] Liu Q.H., Zhang L.J., Wang J., Wu B.W., Cao J.M. (2021). Cardioprotection of an I_K1_ channel agonist on L-thyroxine induced rat ventricular remodeling. Am. J. Transl. Res..

[46] Liu Q.H., Qiao X., Zhang L.J., Wang J., Zhang L., Zhai X.W., Ren X.Z., Li Y., Cao X.N., Feng Q.L. (2019). I_K1_ Channel Agonist Zacopride Alleviates Cardiac Hypertrophy and Failure *via* Alterations in Calcium Dyshomeostasis and Electrical Remodeling in Rats. Front. Pharmacol..

[47] Omole J.G., Alabi Q.K., Aturamu A., Adefisayo M.A., Oluwayomi O., Dada M.B., Ige M.S. (2019). Barium chloride dose-dependently induced heart and lung injury in Wistar rats. Environ. Toxicol..

[48] Heijman J., Algalarrondo V., Voigt N., Melka J., Wehrens X.H., Dobrev D., Nattel S. (2016). The value of basic research insights into atrial fibrillation mechanisms as a guide to therapeutic innovation: A critical analysis. Cardiovasc. Res..

[49] Árpádffy-Lovas T., Mohammed A.S.A., Naveed M., Koncz I., Baláti B., Bitay M., Jost N., Nagy N., Baczkó I., Virág L. (2022). Species-dependent differences in the inhibition of various potassium currents and in their effects on repolarization in cardiac ventricular muscle. Can. J. Physiol. Pharmacol..

[50] Mezentseva L.V., Kashtanov S.I., Vostrikov V.A., Zviagintseva M.A., Kosharskaia I.L. (2002). Analiz EKG pri fibrilliatsii zheludochkov u cheloveka i zhivotnykh na osnove teorii khaosa [Analysis of ECG in ventricular fibrillation in man and animals based on chaos theory]. Biofizika.

[51] Kaese S., Frommeyer G., Verheule S., van Loon G., Gehrmann J., Breithardt G., Eckardt L. (2013). The ECG in cardiovascular-relevant animal models of electrophysiology. Herzschrittmachertherapie Elektrophysiologie.

[52] Sharma A.K., Singh S., Bhat M., Gill K., Zaid M., Kumar S., Shakya A., Tantray J., Jose D., Gupta R. (2023). New drug discovery of cardiac anti-arrhythmic drugs: Insights in animal models. Sci. Rep..

[53] Dobrev D., Wehrens X.H.T. (2018). Mouse Models of Cardiac Arrhythmias. Circ. Res..

[54] Zimmer T., Haufe V., Blechschmidt S. (2014). Voltage-gated sodium channels in the mammalian heart. Cardiol. Cardiol. Sci. Pract..

[55] Clauss S., Bleyer C., Schüttler D., Tomsits P., Renner S., Klymiuk N., Wakili R., Massberg S., Wolf E., Kääb S. (2019). Animal models of arrhythmia: Classic electrophysiology to genetically modified large animals. Nat. Rev. Cardiol..

